# Identification of Bioactivity, Volatile and Fatty Acid Profile in Supercritical Fluid Extracts of Mexican arnica

**DOI:** 10.3390/ijms17091528

**Published:** 2016-09-12

**Authors:** J. Saúl García-Pérez, Sara P. Cuéllar-Bermúdez, Alejandra Arévalo-Gallegos, José Rodríguez-Rodríguez, Hafiz M. N. Iqbal, Roberto Parra-Saldivar

**Affiliations:** Tecnologico de Monterrey, Campus Monterrey, Ave. Eugenio Garza Sada 2501, 64849 Monterrey, N.L., Mexico; garcia.saul@itesm.mx (J.S.G.-P.); sara.cuellarbermudez@kuleuven.be (S.P.C.-B.); alejandra.arevalog@itesm.mx (A.A.-G.); jrr@itesm.mx (J.R.-R.); hafiz.iqbal@tecmx.onmicrosoft.com (H.M.N.I.)

**Keywords:** supercritical-CO_2_, Mexican arnica, *Heterotheca inuloides*, antioxidant, paper-disc difussion test, fatty acid, sesquiterpenoids

## Abstract

Supercritical fluid extraction (SFE) is a sustainable technique used for the extraction of lipophilic metabolites such as pigments and fatty acids. Arnica plant is considered a potential candidate material with high antioxidant and antimicrobial activities. Therefore, in this study, a locally available *Heterotheca inuloides*, also known as Mexican arnica, was analyzed for the extraction of high-value compounds. Based on different pressure (P), temperature (T), and co-solvent (CoS), four treatments (T) were prepared. A maximum 7.13% yield was recovered from T2 (T = 60 °C, P = 10 MPa, CoS = 8 g/min), followed by 6.69% from T4 (T = 60 °C, P = 30 MPa, CoS = 4 g/min). Some bioactive sesquiterpenoids such as 7-hydroxycadalene, caryophyllene and δ-cadinene were identified in the extracts by GC/MS. The fatty acid profile revealed that the main components were palmitic acid (C16:0), followed by linoleic acid (C18:2ω6c), α-linolenic acid (C18:3ω3) and stearic acid (C18:0) differing in percent yield per treatment. Antibacterial activities were determined by the agar diffusion method, indicating that all the treatments exerted strong antibacterial activity against *S. aureus*, *C. albicans*, and *E. coli* strains. The antioxidant capacity of the extracts was also measured by three in vitro assays, DPPH, TEAC and FRAP, using Trolox as a standard. Results showed high antioxidant capacity enabling pharmaceutical applications of Mexican arnica.

## 1. Introduction

A wider spectrum of naturally occurring plants with medicinal value has been utilized for thousands of years in traditional medicine. Among them, *Heterotheca inuloides*, (known as Mexican arnica) has attracted a great deal of interest due to its potent characteristics as anti-inflammatory agent, as anti-microbial and anti-oxidant, and as a rich source of high-value compounds [1]. *H. inuloides* belongs to the Asteraceae family, it is a herbaceous perennial plant and is widely distributed in the mountainous areas in Mexico [2]. Owing the above-mentioned novel characteristics, Mexican arnica is used in phyto-preparations for tropical treatment purposes, such as contusions, sprains and rheumatic disorders [3]. Bioactive components exhibiting pharmacological effects in arnica are mainly sesquiterpene lactones and their ester derivatives. Its anti-inflammatory properties are due to the presence of sesquiterpene lactones [4], while its anti-microbial activity and anti-oxidant capacity are attributed to phenolic compounds and flavonoids that act synergistically with the sesquiterpene lactones [5].

Extraction of high-value compounds from medicinal plants has been reported mainly by solvent extraction [6], whereas, these processes are undesirable because of the presence of residual solvent in the extract. However, in past few years, new methodologies have been developed/proposed for the quickest and efficient extraction of high-value compounds from different materials. Among them, SC-CO_2_ fluid extraction is the most promising technique for extraction of naturally occurring bioactive compounds. Moreover, SC-CO_2_ fluid extraction allows a higher extraction selectivity and low degradability without the use of non-food grade solvents [7].

Supercritical fluids feature low viscosity and high diffusivity of the fluid that are essential for an efficient extraction of desirable compounds [8]. Carbon dioxide (CO_2_) is the most used fluid for this purpose because it can reach high solvating capacity, it is widely available, relatively inexpensive and secure [9]. Compared with organic solvents, supercritical-carbon dioxide (SC-CO_2_) does not produce hazardous residues, avoiding purification and further solvent recovery [10]. The employment of a supercritical extract of *H. inuloides* is promising, different parameters such as yield and composition should be taken into account. The aerial parts of *H. inuloides* have long been used as a folk medicine for the treatment of several disorders, such as postoperative thrombophlebitis and used externally as a remedy for acne, bruises and muscle aches [11]. Currently in the market, a semi-solid preparation with supercritical extract of *A. montana* is being used because of its anti-inflammatory properties [12]. In this work, Mexican arnica (*Heterotheca inuloides*) extract was obtained by means of SC-CO_2_ fluid extraction. So far, there is none or few reports available on SC-CO_2_ fluid extraction for Mexican arnica. To the best of our knowledge, the bioactivity profile of Mexican arnica (*Heterotheca inuloides*) plant extract by SC-CO_2_ is described for the first time in this study. Also, its anti-oxidant and anti-microbial activities were also investigated. Finally, fatty acid composition of this indigenous arnica extract was analyzed by spectrophotometric assays and gas chromatography (GC). 

## 2. Results and Discussion

### 2.1. Extraction Yield

The operational conditions used for SFE and the obtained yields are shown in Table 1. Supercritical extracts were yellowish liquids with percent yield ranging from 4.62% to 7.13% (*w*/*w*). In this study we used a Taguchi design (L4) to analyze the main factors affecting the supercritical extraction of *H. inouloides*: pressure (100, 30 MPa), temperature (40, 60 °C) and co-solvent flow (4 and 8 g/min). Ethanol was chosen as a co-solvent due to its GRAS (Generally Recognized As Safe) status and because of its high polarity (5.2), solubilizing a wide range of polar compounds [13]. 

**Figure 1:**
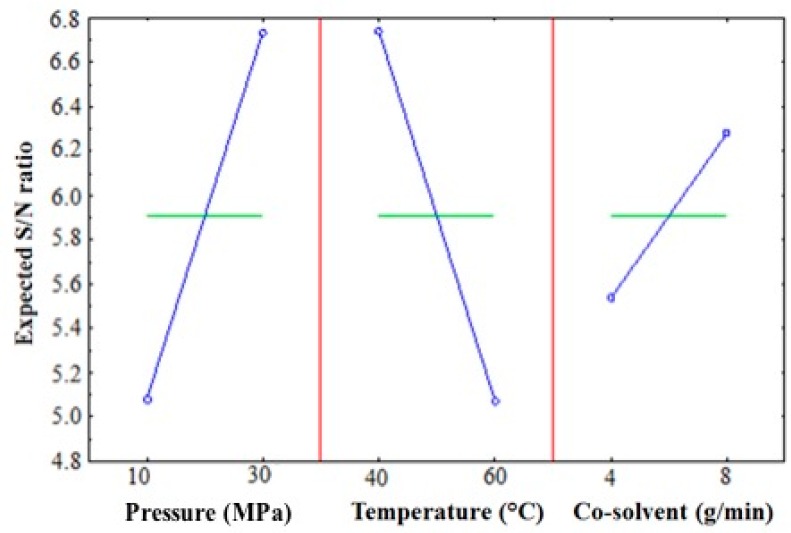
Results of the Taguchi experiment to identify the most influential factors on the extraction yield (%) of Mexican arnica. Higher signal to noise ratio (S/N) indicates higher influence on the extraction yield by means of supercritical CO_2_. Blue lines represent the size of the effect, green lines represent the optimal S/N ratio.

Four different treatment conditions were applied and the highest extraction yield (7.13% *w*/*w*) was the result of a combination of high pressure (30 MPa), low temperature (40 °C) and high co-solvent flow (8 g/min), which is also favorable since high pressure contributes to cell rupture, facilitating compounds extraction [14]. The low temperature additionally helps against thermal degradation of sesquiterpenes, the main bioactive elements of *H. inuloides* [15]. The analysis of variance (ANOVA) showed that pressure and temperature are factors with statistical significance (*p* < 0.1) with regards to the extraction yield (Table 2). All the evaluated treatments were further analyzed through Taguchi design and the results obtained are illustrated in Figure 1. 

### 2.2. Gas Chromatography (GC) Analysis 

The previously extracted samples were re-dissolved in a mixture of ethanol and dichloromethane (7:3) prior to the chromatographic analysis. The resultant compounds obtained by gas chromatography-mass spectrometry (GC-MS) analysis are summarized in Table 3. A total of 15 compounds were identified, among them some fatty acid decarboxylation products. Previous reports by solvent extraction of *H. inuloides* have shown cytotoxic effects against breast carcinoma (BT-20 cells), epitheloid cervix carcinoma (HeLa cells), and melanoma skin in human and mouse cells [16]. Some bioactive sesquiterpenoids identified in such Mexican arnica extracts are 7-hydroxy-3,4-dihydrocadalene, 7-hydroxycadalene, β-caryophyllene and β-caryophyllene epoxide [17]. As expected, these compounds were also present in our extracts, confirming the potential application of Mexican arnica in pharmaceutical applications. 

### 2.3. Fatty Acid Profile

**Figure 2:**
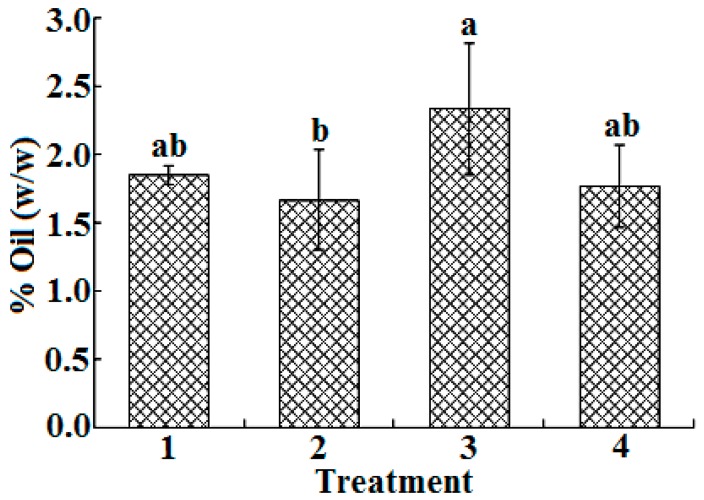
Total percentages of triglycerides presented in supercritical extracts of Mexican arnica. Statistical difference between treatments is denoted by letters a and b.

The total percentage of triglycerides present in the supercritical extracts of *H. inuloides* are shown in Figure 2, ranging from 1.7% to 2.3% (*w*/*w*). Previous reports on *H. inuloides* have investigated the presence of compounds such as flavonoids, sesquiterpenoids and sterols, but a few studies have investigated the presence of the lipid fraction [18]. Essential fatty acids have been considered as nutraceuticals and functional foods, playing a key role in scientific research. Moreover, they also play a significant role in a range of biochemical pathways, and therefore possess useful medicinal properties such as cardio-protective and anti-inflammatory, among others [19]. 

Table 4 illustrates the fatty acid profile of Mexican arnica extracts. The main components were palmitic acid (C16:0), followed by linoleic acid (C18:2ω6c), α-Linolenic acid (C18:3ω3) and stearic acid (C18:0), varying in their percent availability per treatment. Fatty acids are skin penetration enhancers, improving the penetration of bioactive anti-inflammatory compounds such as sesquiterpenoids present in the extracts [20]. Soxhlet extraction performed in *H. inuloides* reported a fatty acid profile with high concentration of eicosatetraenoic n-3 (20:4), *cis*-9-hexadecenoic n-7 (C16:1), hexacosanoic (C20:0) and *cis*-9-octadecenoic acid (C18:1) [18], the differences between the profile obtained by Soxhlet and the obtained in this study could be explained because of the varying arnica growth or culture conditions, along with the effect of solvent polarity in the extraction by selectivity of supercritical CO_2_ compared with Soxhlet extraction [21]. 

### 2.4. Antimicrobial Susceptibility Test

**Figure 3:**
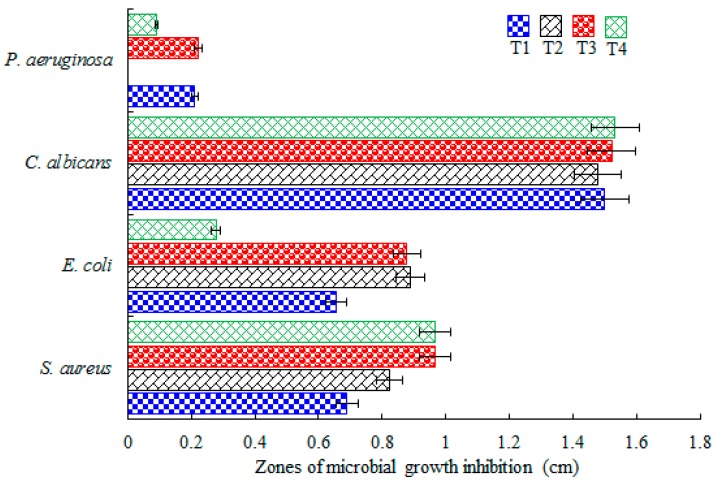
Antibacterial activities of four different treatments of Mexican arnica extracts against *S. aureus*, *C. albicans*, *P. aeruginosa* and *E. coli* strains. Mean area of the zones of microbial growth inhibition in cm (*n* = 3).

The antibacterial and antifungal activity of supercritical extracts was tested against *Staphylococcus aureus* ATCC 25923, *Candida albicans* ATCC 10231, *Pseudomonas aeruginosa* ATCC 27853 and *Escherichia coli* ATCC 25922. It was observed that all tested treatments exhibited antimicrobial behavior against *S. aureus* ATCC 25923 which is among the most common Gram positive bacteria causing food poisoning. As it is shown in Figure 3, all four treatments (T1 to T4) showed a strong bactericidal effects against *C. albicans* ATCC 10231, whereas in case of *E. coli* ATCC 25922 only T1, T2 and T3 were among the most effective treatments. By contrast, T4 was not as effective particularly against *E. coli* ATCC 25922 and *P. aeruginosa* ATCC 27853. None of the Mexican arnica extracts demonstrated a clear and considerable zone of inhibition against *Pseudomona aeruginosa* ATCC 27853.

### 2.5. Antioxidant Capacity

Antioxidant molecules can be divided into preventive antioxidants and chain-breaking antioxidants. The former are responsible for scavenging reactive oxygen species (ROS) and the latter react with chain-propagating radicals [22]. The main constituents of *H. inuloides* responsible for its antioxidant properties are flavonoids, classified as preventive antioxidants, and sesquiterpenes, recognized as chain-breaking antioxidants [23]. Its antioxidant capacity has been studied via a series of peroxidation and scavenging assays: its inhibitory activity against lipid peroxidation in liver microsomes, red blood cells and mitochondria [24]. A treatment with a methanolic extract of *H. inuloides* decreased the activity of antioxidant enzymes such as catalase and Gpx in induced liver injuries [25] and its scavenging activity has been proven in several assays including OH˙ scavenging capacity, singlet oxygen, HOCl and ONOO-scavenging assays [1]. These studies used extracts obtained via solvent extraction, with either acetone or methanol. To the best of our knowledge, there is no information reported on antioxidant activity assays performed on supercritical *H. inuloides’* extracts.

**Figure 4:**
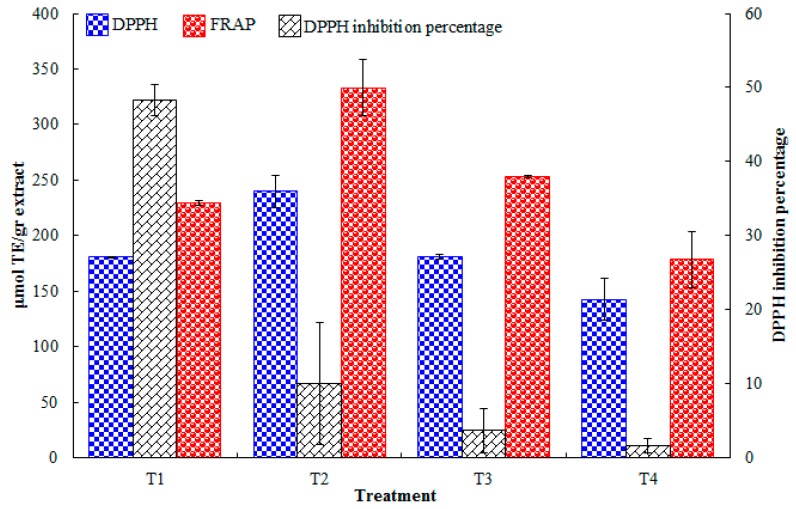
Scavenging activity of the supercritical *H. inuloides* extracts determined by the 2,2-diphenyl-1-picrylhydrazyl (DPPH) and Ferric Reducing Antioxidant Power (FRAP) assays. Results are expressed in DPPH inhibition percentage (primary vertical axis) and µmol Trolox Equivalents (TE)/g of extract (secondary vertical axis).

The DPPH assay is based on the antioxidants’ ability to quench the DPPH· radicals and while monitoring both single electron and hydrogen atom transfer mechanisms [26]. Figure 4 shows the inhibition percentage of the supercritical extracts obtained from each extraction treatment. T1 (10 MPa, 40 °C, 4 g/min co-solvent) showed the highest inhibition percentage at almost 50%. The results from the DPPH and FRAP assays are expressed in terms of µmol Trolox Equivalents (TE)/g of extract. Overall, T2 (10 MPa, 60 °C, 8 g/min co-solvent) yielded the highest antioxidant capacity, followed by T3 (30 MPa, 40 °C, 8 g/min). These results indicate that a higher amount of co-solvent aided the extraction of antioxidant compounds. The statistical analysis determined co-solvent as the most relevant factor, followed by pressure. The optimum treatment was determined to have the highest levels of all factors: 30 MPa, 60 °C and 8 g/min of ethanol. Another study analyzed (by the DPPH assay) the isolated compounds responsible for *H. inuloides’* properties; the results indicate that the flavonoids quercetin and kaempferol and the sesquiterpenes 7-hydroxy-3,4-dihydrocadalin and 7-hydroxycadalin were the most efficient scavengers [22]. Given the polarity of these molecules, it is plausible that co-solvent played such an important role in obtaining highly antioxidant extracts.

## 3. Materials and Methods

### 3.1. Biological Material

All biological materials used in this study were purchased from the downtown supermarket, in Guadalajara, Mexico. The final water content in samples was 3.68% ± 0.1% (*w*/*w*). All collected samples were sieved, washed with hot water (to avoid any dust contamination), and stored in polyethylene bags to prevent the penetration of moisture. The fractions with the particle size <1.41 mm (Mesh No. 14, US Standard) were used for the extraction purposes. 

### 3.2. SC-CO_2_ Extraction and Design of Experiments (DOE)

Extractions were carried out in a pilot-scale plant for supercritical fluid extraction (Biomex, Guadalajara, Mexico) with a 100 mL extraction cell (Waters Thar SFC SFE 100 Equipment, Milford, CT, USA). The Statistica^®^ software (StatSoft Inc., Tulsa, OK, USA) was used for the DOE and results analysis; comparisons showing a *p*-value <0.10 were considered significantly different. For media comparison the Fisher Least Significance Difference (LSD) test was used. Taguchi design was used to analyze the factors of temperature, pressure and co-solvent flow. Two levels of each factor were tested as shown in Table 1. The extraction time was defined as one hour. A fixed CO_2_ flow of 25 g/min was used for all extractions, resulting in a S:F ratio of 150. Initial 40 min of the extractions were performed along with co-solvent flow. The last 20 min of the extraction were performed with just the CO_2_ flow in order to allow for a proper drying of the sample. All treatments were performed in triplicate.

### 3.3. Gas Chromatography (GC) Analysis 

A screening of compounds in the extracts was made by gas chromatography (GC) (7890B, Agilent Technologies, Santa Clara, CA, USA) connected to a mass spectroscopy detector (MS) (5977A, Agilent Technologies). Separation was carried out on a capillary column HP-5 MS (5% phenyl/95% dimethylpolysiloxane), size of 30 m × 0.25 mm × 0.25 μm (J&W Scientific, Santa Clara, CA, USA) with helium as a carrier gas with a flow of 1 mL/min. The injection was performed with a split ratio 15:1 at 270 °C. The ramp temperature in the oven started at 70 °C (1.0 min), followed by an increase to 200 °C (10 °C/min) and a holding period of 1 min, and by an increase to 310 °C (10 °C/min) and a final holding period of 7 min. The ion source and mass quadrupole temperature were 150 and 250 °C, respectively. The ionization was carried out with an electronic impact source at 70 eV. The mass range monitored by the analyzer was 30–350 Uma. The compounds were identified according to the NIST (National Institute of Standards and Technology) database. A match criterion of >85% was applied. 

### 3.4. Fatty Acid Determination 

All chemical substances were HPLC (High Performance Liquid Chromatography) grade. Solvents and acids were purchased from Sigma-Aldrich (Toluca, Mexico). Solvent systems during the test were: system A (hexane:acetone; 80:20 *v*/*v%*), system B (Methanol:Sulfuric Acid; 93:7 *v*/*v*%), and system C (hexane). Undecanoic fatty acid was used as an internal standard (400 mg/L, Supelco Inc., Bellefonte, Pennsylvania, PA, USA). A multi-standard FAME mix (Fatty Acid Methyl Esters, 10 mg/mL, Supelco Inc.) was used for fatty acid identification. 2 mL of a sample, 400 µL of internal standard solution and 2 mL of system B were heated to 80 °C for 1 h for transesterification. Once at room temperature, 5 mL (×2) of system C were added and mixed for 1 min. The upper phase was transferred for gas chromatography (GC) analysis, with a flame ionization detector (GC/FID 6890 Series Agilent Technologies, Santa Clara, CA, USA), equipped with a SP™-2380 column (30 m, 0.25 mm d (diameter), 0.20 μm of film). The temperature ramp in the oven started at 50 °C (1 min), followed by an increase up to 240 °C (4 °C/min) and a holding period of 4 min. The injector was operated at 260 °C, while the detector at 280 °C. Nitrogen gas was used as a carrier gas. 

### 3.5. Antimicrobial Susceptibility Test

The antimicrobial activity of all the extracts was evaluated by a paper-disc diffusion test against *Staphylococcus aureus* ATCC 25923, *Candida albicans* ATCC 10231, *Pseudomona aeruginosa* ATCC 27853, *Escherichia coli* ATCC 25922 strains. An inoculum of the above-mentioned strains (1.5 × 10^8^ CFU/mL, 0.5 Mcfarland Standard, BD Inc., Franklin Lakes, NJ, USA) was applied to the surface of a Mueller-Hinton agar plate (20 mL, BD Inc.). Filter papers (5 mm, Whatman 1, GE Healthcare, Little Chalfont, UK) were saturated in 1 mL of the different extract solutions. Six filter paper disks were placed on the inoculated agar surface. The filter paper disks fed with solvent alone were considered as a control sample. The zones of growth inhibition around the disks were measured after 24 h of incubation at 35 °C, except for *C. albicans*, for which samples were incubated at 25 °C for 24 h. The sensitivities of the test strains to the plant extracts were determined by measuring the sizes of inhibitory zones (including the diameter of disk) on the agar surface around the disks. Values <6 mm were considered as not active against each test strain. All tests were performed in triplicate and ampicillin was used as a positive control. 

### 3.6. Antioxidant Capacity

2,2-Diphenyl-1-picrylhydrazyl (DPPH), 2,2′-azino-bis(3-ethylbenzothiazoline-6-sulphonic acid (ABTS), Trolox, sodium acetate tri-hydrate, ferric chloride, and 2,4,6-Tripyridyl-s-Triazine (TPTZ) were purchased from Sigma-Aldrich, Toluca, Mexico. All other solvents were of analytical laboratory grade. Aliquots of 1 mL were taken from each SFE extract. The solvent was evaporated under nitrogen and then the sample was dissolved in methanol and stored at −80 °C until antioxidant assays. 

#### 3.6.1. 2,2-Diphenyl-1-picrylhydrazyl (DPPH) Radical Scavenging Assay

The DPPH assay was done according to the method of Brand Williams et al. [27], with some modifications. A 60 µM DPPH working solution was prepared by dissolving 11.83 mg DPPH in 500 mL methanol. A 75 µL aliquot of this extract was allowed to react with 3 mL of the DPPH solution for 4 h in the dark. Following that, the absorbance was measured at 516 nm wavelength. The antioxidant activity of the extracts was calculated as (i) µM TE/g extract, using a standard curve between 100 and 1000 µM Trolox; and (ii) inhibition (%) of DPPH absorbance = ((A_control_ − A_test_)/A_control_) × 100.

#### 3.6.2. Ferric Reducing Antioxidant Power (FRAP)

The FRAP assay was done according to Benzie and Strain [28], with some modifications. The FRAP assay is based on the reduction of the colorless ferric complex (Fe^3+^-tripyridyltriazine) to the blue colored ferrous complex (Fe^2+^-tripyridyltriazine) by the presence of electron-donating antioxidants. The reduction was monitored by taking absorbance at 593 nm. The FRAP reagent was prepared daily by mixing 10 volumes of 300 mM acetate buffer, pH 3.6, with 1 volume of 20 mM ferric chloride and 1 volume of 10 mM TPTZ in 40 mM HCl and warmed at 37 °C before use. About 100 µL of methanolic extract was allowed to react with 3 mL FRAP solution. Results were expressed in µM TE/g extract. 

#### 3.6.3. ABTS Antioxidant Assay

The ABTS method described by Luis et al. [29] was performed based on the reduction of ABTS^+^ radicals by antioxidants present in the sample. ABTS^+^ radical cation was produced by mixing 7 mM ABTS with 2.45 mM potassium persulfate in PBS (phosphate buffer saline, pH 7.4), and incubating the mixture in the dark at room temperature for 16 h. Next, the ABTS^+^ solution was diluted with PBS to an absorbance of 0.70 at 734 nm. About 10 µL of methanolic extract was added to 1 mL of diluted ABTS^+^ radical solution. The absorbance was taken 6 min after initial mixing at 734 nm. A calibration curve was established using Trolox as a reference standard and results were expressed in µM TE/g extract.

## 4. Conclusions

The supercritical extract has shown optimal yield conditions of P = 30 MPa, T = 40 °C and Co-solvent flow of 8 g/min. Regarding the composition, sesquiterpenoids were detected by GC/MS, because they are the main bioactive molecules in the plant. The fatty acid profile showed higher amounts of palmitic acid (C16:0), linoleic acid (C18:2ω6c), α-linolenic acid (C18:3ω3) and stearic acid (C18:0), exhibiting anti-inflammatory, skin penetration enhancement and cardio-protective properties. In this work we demonstrate the potential applications of *H. inuloides* supercritical extracts for medical applications as antioxidant, antimicrobial and as a rich source of high value compounds and fatty acids. However, further studies must assess cytotoxic effects before scale-up commercialization. 

## Figures and Tables

**Table 1:** Supercritical fluid extraction (SFE) conditions used for extractions and obtained yields.

Treatment	T (°C)	P (MPa)	EtOH^+^ (g/min)	Extracted Mass (g)	Yield (%)
1 ab	40	10	4	0.55 ± 0.03	5.54 ± 0.30
2 a	60	10	8	0.71 ± 0.02	7.13 ± 0.27
3 b	40	30	8	0.46 ± 0.08	4.62 ± 0.87
4 a	60	30	4	0.67 ± 0.14	6.69 ± 1.44

^+^Ethanol 96% was used as co-solvent, fixed conditions were CO_2_ flow (25 g/min), extraction time of 60 min (40 min CO_2_ plus Co-solvent + 20 min with only CO_2_). All treatments were performed in triplicate. Statistical difference between treatments is denoted by letters a and b.

**Table 2:** Analysis of variance of the different treatments of Mexican arnica extracted with supercritical CO_2_.

Parameter	SS	df	MS	*F*	*p*-Value
Pressure	8.20	1	8.20	4.10	0.077
Temperature	8.32	1	8.32	4.17	0.075
Co-solvent	1.66	1	1.66	0.83	0.388
Residual	15.97	8	1.99		

SS: Sum of squares; df: Degrees of freedom; MS: Mean squares; F: F ratio.

**Table 3:** Gas chromatography-mass spectroscopy (GC-MS) composition of the different treatments of Mexican arnica supercritical extracts. The relative areas are shown in percent. Statistical difference between treatments is denoted by letters a and b.

Compound	MW * (g/mol)	Relative Area (%) Per Treatment			
		1	2	3	4
4(3H)-Quinazolinone, 7-amino-3-ethyl–	189.09	-	-	-	2.732
7-Hydroxycadalene	214.13	5.05 a	-	4.913 a	4.969 a
Cadalene	198.14	10.34 a	8.755 b	9.520 ab	9.709 ab
Caryophyllene	204.18	1.35 a	1.084 a	1.117 a	1.087 a
Caryophyllene oxide	220.18	4.08 a	3.494 a	3.441 a	4.131 a
Docosane	310.36	-	10.185 a	8.926 a	-
Eicosane	282.32	2.09 a	-	3.363 a	2.202 a
Heneicosane	296.34	-	5.653 a	2.703 b	-
Hexacosane	366.42	19.53 a	8.685 b	17.616 a	14.146 b
Nonacosane	408.47	8.61 a	8.770 a	-	-
Octadecane	254.29	-	1.443	-	-
Octadecane, 1-iodo–	380.19	-	-	3.534 a	4.668 a
Pentacosane	352.40	2.67 a	2.954 a	1.701 a	1.540 a
Spathulenol	220.18	-	-	-	0.603
δ-Cadinene	204.18	2.11 a	1.879 b	1.904 b	1.913 b

* MW = Molecular Weight; Statistical difference between treatments is denoted by letters a and b.

**Table 4:** Fatty acid profiles presented in arnica supercritical extracts. Results are presented in percentage (*w*/*w%*).

Fatty Acid	Fatty Acid Percentage (%) Per Treatment			
	T1	T2	T3	T4
Lauric C12:0	0.5 ± 0.0	-	-	-
Myristic C14:0	2.4 ± 0.0	2.4 ± 0.01	2.6 ± 0.01	2.4 ± 0.01
Palmitic C16:0	20.6 ± 0.02	21.4 ± 0.07	22.2 ± 0.12	21.2 ± 0.06
Palmitoleic C16:1	1.2 ± 0.01	1.0 ± 0.0	1.1 ± 0.01	1.5 ± 0.01
Stearic C18:0	7.6 ± 0.01	7.3 ± 0.03	8.5 ± 0.04	7.5 ± 0.02
Oleic C18:1ω9c	5.0 ± 0.01	5.1 ± 0.01	5.4 ± 0.03	5.4 ± 0.0
Linolelaidic C18:2ω6t	3.8 ± 0.02	2.5 ± 0.01	3.3 ± 0.02	2.9 ± 0.02
Linoleic C18:2ω6c	13.6 ± 0.01	16.4 ± 0.06	16.1 ± 0.08	15.5 ± 0.05
γ-Linolenic C18:3ω6	1.8 ± 0.0	1.6 ± 0.01	1.9 ± 0.01	1.7 ± 0.01
α-Linolenic C18:3ω3	7.0 ± 0.0	7.6 ± 0.03	7.5 ± 0.04	7.3 ± 0.03
Behenic C22:0	2.5 ± 0.0	2.3 ± 0.01	2.6 ± 0.01	2.4 ± 0.01
Erucic, C22:1ω9	1.7 ± 0.0	3.3 ± 0.01	2.5 ± 0.01	2.2 ± 0.01
Lignoceric, C24:0	4.2 ± 0.01	3.9 ± 0.02	4.7 ± 0.03	4.2 ± 0.02
